# Multiparametric MRI for Localized Prostate Cancer: Lesion Detection and Staging

**DOI:** 10.1155/2014/684127

**Published:** 2014-11-30

**Authors:** Daniel J. A. Margolis

**Affiliations:** David Geffen School of Medicine at UCLA, 200 Medical Plaza, No. 165-43, MC 695224, Los Angeles, CA 90095-6952, USA

## Abstract

Multiparametric MRI of the prostate combines high-resolution anatomic imaging with functional imaging of alterations in normal tissue caused by neoplastic transformation for the identification and characterization of *in situ* prostate cancer. Lesion detection relies on a systematic approach to the analysis of both anatomic and functional imaging using established criteria for the delineation of suspicious areas. Staging includes visual and functional analysis of the prostate “capsule” to determine if *in situ* disease is, in fact, organ-confined, as well as the evaluation of pelvic structures including lymph nodes and bones for the detection of metastasis. Although intertwined, the protocol can be optimized depending on whether lesion *detection* or *staging* is of the highest priority.

## 1. Introduction

The principles behind prostate imaging, specifically anatomic and functional imaging, have been described in earlier articles in this special issue, and subsequent articles will address the use of multiparametric prostate magnetic resonance imaging (mpMRI) for, among other things, image-guided biopsy, active surveillance, and focal therapy planning, as well as the principles of standardized reporting. This paper will focus on the detection and staging of prostate cancer as identified on mpMRI.

In order to understand the relevance of mpMRI for prostate cancer detection, it is important to review the treatment choice for treating prostate cancer, as mpMRI is increasingly being used to choose between these options [1–6]. Treatment for prostate cancer has traditionally been based largely on clinical factors: the serum prostate specific antigen (PSA) level and PSA density; the grade, length, and number of positive prostate biopsies; and the digital rectal exam (DRE) [7]. Treatment options include active surveillance (deferring definitive management for low-grade, small-volume disease), surgery, radiation therapy, and hormone modulation. Focal therapy is emerging as yet another treatment strategy, with its own guidelines. Even within these broad categories, subtle differences exist. The open radical retropubic prostatectomy has been largely replaced by robotic prostatectomy, for which improved outcomes have recently been shown [8]. Within radiation therapy, patients and physicians can now make the choice between conformal external beam radiation therapy, brachytherapy permanent seed implantation, and high dose rate brachytherapy temporary seed implantation.

Currently, prostate cancer detection is largely qualitative, based on the appearance on T2-weighted imaging (T2WI), diffusion-weighted imaging (DWI), and dynamic contrast-enhanced (DCE) perfusion imaging, as well as, when available, magnetic resonance spectroscopic imaging (MRSI). The cues to identify cancer and how to avoid pitfalls are compiled here but have been described elsewhere [9–12]. Once a region is identified as suspicious for cancer, it must be determined whether or not it is organ-confined. Whether the tumor is organ-confined (stage T2) or has extended outside of the prostate capsule (stage T3a) or into seminal vesicles (stage T3b) is of utmost importance for surgical planning and may determine which of the myriad of treatment choices are appropriate for the patient.

## 2. Lesion Detection and Staging Core Concepts

Because of its small size and deep location, optimizing signal to noise (SNR) of magnetic resonance images at the level of the prostate is challenging. The development of the endorectal coil, much to the rue of our patients, resulted in the 10-fold improvement in SNR over transabdominal phased-array acquisition. With current gradient performance at 3.0 T, however, it is controversial how much value the endorectal coil still adds. Its main value may be in resolution of the prostate capsule and the ability to acquire MRSI and high* b*-value DWI, although artifacts such as geometric distortion and susceptibility are worse at high field strength [13–15].

High matrix, reduced field-of-view imaging is also important for resolution of prostate tumors. A volume of 0.5 cm^3^, which is considered significant, corresponds to a diameter of 1 cm, so tumors of this size must be evaluable [16]. The parameters recommended by the European Society of Urogenital Radiology (ESUR) are due to be updated this year jointly with the American College of Radiology and outline optimal technical specifications [17]. A summary of recommendations of the 2012 guidelines is given in Table 1.

Other recommendations are for an antiperistaltic agent such as hyoscine butylbromine (Buscopan) or glucagon and an at least 8-channel external phased array regardless of the use of an endorectal coil. The antiperistaltic agent reduces motion artifacts. An optimized external phased-array coil matrix ensures optimal SNR. Optimizing image quality is essential for accurate detection and staging.

## 3. Lesion Detection

### 3.1. Protocol Considerations

Although the general recommendations for pulse sequences from the ESUR are outlined in Table 1, the important considerations for lesion detection are optimal image quality of both T2WI and functional imaging—DWI, DCE, and (if acquired) MRSI. As mentioned above, an endorectal coil is not absolutely necessary, but the utility will depend on the performance of the scanner in question. Signal efficiency characteristics will determine factors such as the number of excitations (signal averages) for low-SNR pulse sequences such as DWI and MRSI. Patient size may also be a factor; the distance from the external phased array to the prostate increases with increasing abdominal girth, such that SNR losses in larger patients may necessitate consideration for an endorectal coil.

For detection alone, however, the protocol can be tailored to limit the amount of time the patient is in the scanner. Axial small field-of-view imaging is nearly imperative, but other than a sagittal localizer additional planes (e.g., true fast or turbo spin-echo coronal and sagittal) for T2WI may be omitted. If the acquisition is 3-dimensional and isovolumetric, the additional planes can be reconstructed with nearly no loss of image quality. However, 3-dimensional acquisitions often take twice as long as the corresponding 2-dimensional imaging such that they may be more time-efficient. Full pelvis large field-of-view imaging is also unnecessary for detection of intraprostatic disease. Limited field-of-view DWI may also improve geometric distortion [18]. Although focusing on the prostate only may not save much time in terms of DCE, it does mean that the decreased coverage of the rest of the pelvis can be spent on either temporal or spatial resolution. If the endorectal coil is not used, MRSI is normally also deferred, resulting in additional time savings.

The review of images is also an important consideration. Being able to view all of the parameters spatially coregistered is integral to multiparametric analysis. Although most PACS software programs allow for spatial coregistration and linking it is equally important to consider that slight motion between acquisitions can result in misregistration so visual inspection for such should be undertaken before attempting to detect focal abnormalities.

The use of mpMRI for the detection of prostate cancer has shown consistently high specificity and variably high negative predictive values [19]. Although there has been interest in eschewing DCE because of its added cost, many studies have shown added value of DCE [20]. Its main use may lie in lesion detection rather than characterization as it tends to show stronger sensitivity than specificity.

### 3.2. Multiparametric Approach

Much like any other multisequence diagnostic process, each of the parameters of mpMRI must be evaluated for optimum sensitivity and specificity of detecting prostate cancer. The relative correlation of each parameter with histology has been well investigated [21–23]. The approach, however, varies by reader. Although tumors show many of the same characteristics in the peripheral zone (PZ) and transitional/central zone (TZ), the zones themselves show differing degrees of homogeneity depending on the parameter. Because the PZ normally shows uniformly unrestricted diffusion, tumors in the PZ are most conspicuous on this parameter. The wide variability of diffusion restriction in the TZ from benign changes decreases the conspicuity of tumors there. Characteristics on T2WI are more useful for discrimination of neoplasia from benign changes in the TZ [22, 24].

Although prostate cancer can be infiltrative, resulting in an underestimation of tumor volume [25], the presence of a focal, mass-like abnormality is common among all of the parameters (except MRSI, the resolution of which is too low for morphology characterization). Uniform low signal on T2WI is a hallmark of tumor. The appearance of the shape and borders of the lesion can also give a sense of the level of suspicion. Geographic or wedge-shaped abnormalities, often with indistinct margins, are often indicative of changes related to inflammation or hemorrhage. A round, oval, or irregular shape is more suspicious, especially when the borders are themselves blurred rather than sharp. The appearance of an “encapsulated” nodule with a thin, discrete low signal border outlining a focal abnormality on T2WI is characteristic of prostatic hyperplasia and is often pivotal for distinguishing benign from suspicious findings in the TZ.

**Figure 1:**
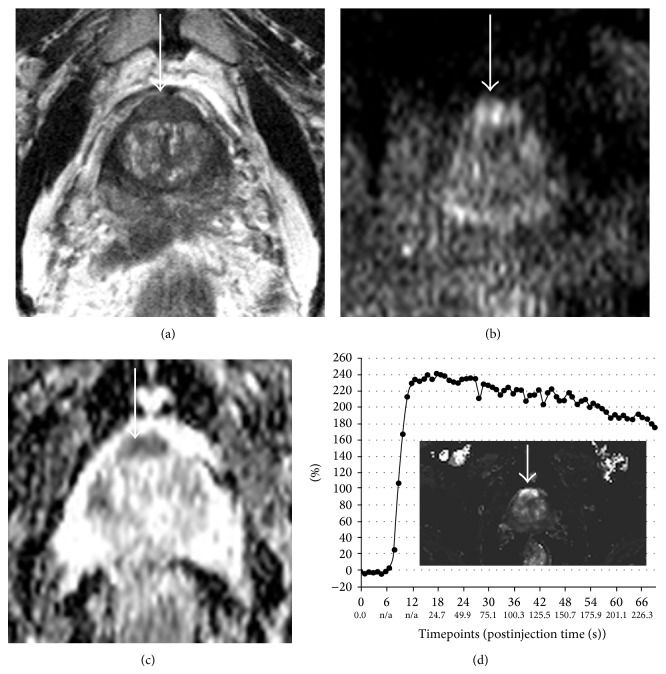
Multiparametric magnetic resonance imaging (mpMRI) detects significant prostate cancer. This 58-year-old man had a doubling of serum PSA in less than 2 years. For image-fusion targeted biopsy planning, mpMRI was performed. An oval, uniformly low signal mass (thick arrow) on T2-weighted imaging (T2WI) with circumscribed margins (a) is associated with focal high signal on diffusion-weighted imaging (DWI) (b) and low signal on the apparent diffusion coefficient (ADC) map (c) signifying restricted diffusion and focal asymmetric enhancement with washout (d) on dynamic contrast-enhanced (DCE) perfusion imaging. Targeted biopsies of this area revealed high volume Gleason 4 + 3 = 7 cancer. Standard systematic or “blind” sextant biopsies, which normally preferentially target the posterior gland, were all negative for any cancer.

The degree to which each of the parameters is abnormal is also a determinant in the level of suspicion. Diffusion-weighted imaging is especially accurate in identifying aggressive disease in the peripheral gland (Figure 1). Because of the inherent heterogeneity of the TZ, functional parameters (especially DCE and DWI) can be variably abnormal in prostatic hyperplasia, such that the T2WI characteristics become much more important in discrimination of truly suspicious areas from likely benign changes. The degree to which DCE and even DWI improve TZ detection remains controversial, possibly related to acquisition parameters [26, 27].

Although extracapsular extension and seminal vesicle involvement are more of an issue for staging than detection, these considerations should not be overlooked even when the indication is detection for biopsy planning. Detecting abnormalities using DCE in the periprostatic space can be challenging given the variability in the density of the venous plexus. The use of T2WI to detect an abnormality arising in the prostate and extending into this space then allows for characterization by functional characteristics. Functional evaluation of the seminal vesicles is also important. Normally, they should be distended with simple fluid, devoid of perfusion or restricted diffusion. When atrophic, the seminal vesicles can appear abnormally dark. However, perfusion is usually overall low and the appearance on the high* b*-value DWI is normally not high signal. The presence of even moderately increased perfusion of restricted diffusion in the seminal vesicles associated with a low signal mass arising in the prostate itself should be considered suspicious for involvement.

### 3.3. Pitfalls and Caveats

Discrimination of abnormalities on mpMRI that result from benign processes as opposed to neoplasia is essential for optimal specificity [12]. Other than prostatic hyperplasia, as mentioned above, hemorrhage, inflammation and resultant fibrosis, and atrophy can all result in T2-shortening and variable changes on functional imaging. Hemorrhage is usually conspicuous by associated T1-shortening on the precontrast T1-weighted images; higher flip-angles may improve conspicuity. Sequela of prostatitis is often characterized by a linear or wedge-shaped morphology. Hormonal therapy and atrophy can also result in uniform T2-shortening and, occasionally, diffusion restriction, making focal abnormalities less conspicuous. In these cases, a focal perfusion abnormality can alert the reader to the potential presence of neoplasia.

Assessment of the overall quality is also important in determining reader confidence. In addition to benign changes which may compromise image quality, rectal distention with the associated susceptibility can markedly degrade DWI (and potentially MRSI, although the endorectal coil often precludes significant gas). Rectal peristalsis may also result in misregistration and image blurring and compromise perfusion mapping. Bulk patient motion should also be considered. The presence of metallic structures in the pelvis can also result in severe artifacts, indicating the need to open up bandwidth and, if possible, scan at a lower field strength (e.g., 1.5 T as opposed to 3.0 T).

## 4. Staging

### 4.1. Protocol Considerations

As mentioned above, the use of an endorectal coil may be essential for adequate resolution of the prostate capsule. It should be noted that the term “capsule” as it applies to the prostate is controversial, as the prostate is lined by only a thin rim of cells which is not normally felt to constitute a true capsule, but this term persists both in the literature and among surgeons. In a relatively thin patient, with no rectal distention, high field strength and gradient performance, and absence of motion, image quality without the endorectal coil may actually surpass that with one and its inherent signal variation. However, the endorectal coil does provide the highest signal possible in the vicinity of the prostate and can also steady the prostate, precluding or limiting rectal motion and distention. Although there is some evidence that local “T” staging can be accurate at 3.0 T without the endorectal coil, most experts and literature support its use [13, 14, 28, 29].

Because spatial resolution, specifically, of the prostate “capsule,” is of the utmost importance, small field-of-view, high matrix T2WI is the most valuable pulse sequence for local “T” staging. Functional parameters improve diagnostic confidence, and the degree to which they are abnormal correlates with the likelihood of T3 disease, but the degree to which it is present is best characterized on T2WI. A 3-dimensional acquisition may also improve resolution of small or subtle irregularity of the prostate margins.

Full field-of-view images should also be included, especially if locoregional treatment, such as external beam radiation therapy, is considered. Generally, standard T1 and T2WI imaging use for characterization of the pelvic bones is sufficient and can usually detect abnormal lymph nodes as well, although a 3-dimensional postcontrast fat-saturated gradient T1-weighted acquisition can be very useful for lymph node evaluation.

### 4.2. Value of Individual Pulse Sequences

**Figure 2:**
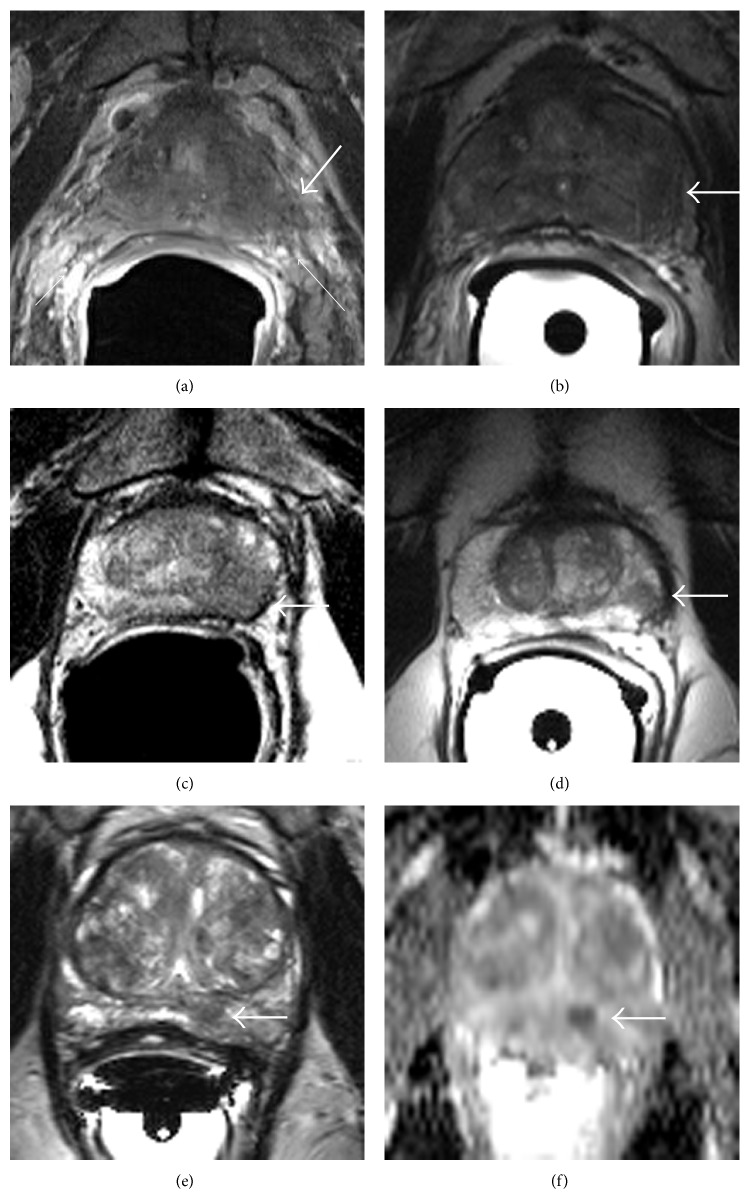
Staging of prostate cancer on T2-weighted imaging. Gross invasion of an irregular, low signal mass (thick arrow) from the prostate to the rectoprostatic angle (thin arrows), the location of the neurovascular bundles (a), which, in the absence of associated hemorrhage, signifies near-certain extraprostatic extension. A low signal mass bulges the capsule (thick arrow) with blurring of the dark line denoting the prostate “capsule” (b), which is high suspicious for at least capsular involvement if not microscopic extraprostatic extension. A low signal mass (thick arrow) with bulging but preservation of this dark line signifying the “capsule” (c) is suspicious for capsular involvement but not frank extraprostatic extension. Focal low signal (thick arrow) which abuts the capsule without a broad base of contact or bulging (d) is low suspicious for capsular involvement. A subtle oval area of low signal (thick arrow) on T2-weighted imaging (e) is confirmed by associated restricted diffusion (f) on the apparent diffusion coefficient map and is not suspicious for capsular involvement or extraprostatic extension. These 5 cases could also be ranked on a 5-point scale, with (a) considered level 5/5 (certain) suspicious for extraprostatic extension and (e) level 1/5 or no suspicious for extraprostatic extension.

Here again we come back to the overwhelming importance of T2WI. Its ability to resolve the margin of the prostate is essential for local “T” staging and detection of extraprostatic extension (EPE) of tumor, especially in terms of informing the surgeon where a patient's EPE lies and whether nerve-sparing surgery can still be considered (Figure 2).

However, functional imaging is also important. Not only does it inform the likelihood that a perceived abnormality on T2WI is aggressive disease, but also greater degrees of functional abnormality have been shown to correlate with increased likelihood of EPE [30–32]. Interestingly, some of these investigations have shown added value of functional imaging not from anatomic localization but simply from the degree to which the parameters are abnormal.

Although metastatic disease detection will be discussed fully in another paper in this special issue, it deserves some mention here. Diffusion-weighted imaging can also be added to whole-pelvis imaging for characterization of lymph nodes and bone lesions [33]. However, a potentially greater improvement in regional spread detection is the use of ultrasmall superparamagnetic iron oxide (USPIO) particles, which are taken up by benign lymph nodes and null their signal. A combination of DWI and USPIO imaging has been shown to be able to detect involvement of normal-sized lymph nodes [34].

### 4.3. Application to Surgical Planning

Pretreatment staging of prostate cancer offers two advantages: improved determination of which patients are not surgical candidates and tailoring the degree to which the neurovascular bundles (NVB) can be spared. Although prostatectomy has shown value even with regional lymph node metastasis and biochemical failure, surgery may be deferred because of the presence of extraprostatic disease. However, the value of extended pelvic lymph node dissection means that regional staging may have more significant implications in presurgical management [35, 36]. Further, mpMRI has shown incremental value in predicting biochemical failure over clinical parameters [37].

In terms of surgical planning, the decision to resect or preserve the neurovascular bundles can have tremendous consequences in the long-term morbidity of the procedure, to the extent that some surgeons will preferentially consider nerve-sparing procedures combined with adjuvant radiation therapy in the presence of positive surgical margins. Surgeons can generally now choose between “interfascial” resection between the prostate “capsule” and NVB and “extrafascial” resection outside of (and therefore sacrificing) the NVB. Robotic technique allows the surgeon an approximated 10-fold improvement in visualization of the surgical field, thus refining these two options. The decision for NVB preservation or resection has traditionally been made based on clinical nomograms.

However, mpMRI has proven value in presurgical planning, which has been established for over a decade [1, 2, 4]. The advantage is that EPE can be determined on a per-bundle basis, whereas the nomograms that rank the risk for EPE do not localize on which side EPE may arise [38]. Preservation of even just one NVB holds the possibility of reduced likelihood for incontinence and impotence over bilateral resection. Even when EPE is detected, if it does not lie near the NVB (e.g., anteriorly) it may be spared entirely.

## 5. Lesion Detection* versus* Staging

### 5.1. Protocol Differences

Since lesion* detection* relies heavily on functional imaging of the prostate only, and lesion* staging* relies on high spatial resolution of the prostate as well as characterization of the remainder of the pelvis, there are opportunities to refine and tailor the protocol to the question at hand. A multiparametric acquisition of two-plane reduced field-of-view T2WI, DWI, and DCE can all be accomplished in well under half an hour with use of an external phased-array coil only, improving patient throughput at the potential cost of sensitivity [39]. Locoregional staging using both small and large field-of-view images and an endorectal coil for improved prostatic resolution will often take longer, although the indications for this protocol may be more limited. For radiation therapy planning, whole-pelvis imaging would be indicated, but the degree to which the prostate capsule is involved is less important; a hybrid protocol, similar to the surgical staging protocol but omitting the endorectal coil, could be considered.

**Figure 3:**
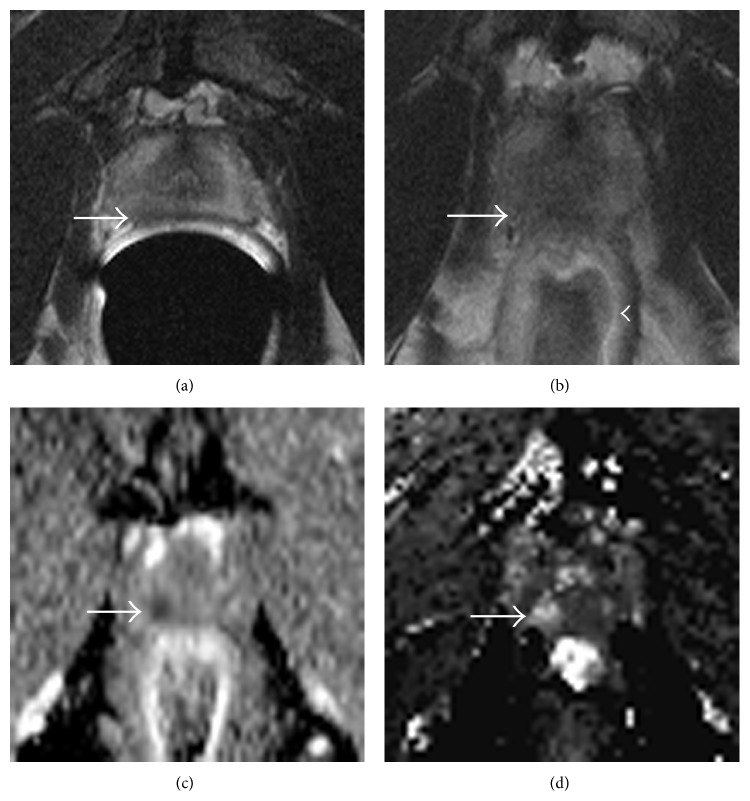
The value of the endorectal coil. A very subtle area of low signal on T2-weighted imaging (a) is associated with blurring of the “capsule” (thick arrow), suggesting capsular involvement. This patient underwent imaging without the coil immediately afterwards (b) where the low signal mass (thick arrow) is indistinct, as is the dark signal line that should signify the “capsule.” As the rectum is distended (arrowhead), this may result from susceptibility artifact. Restricted diffusion on the apparent diffusion coefficient (ADC) map (c) and focal increased perfusion on the dynamic contrast-enhanced (DCE) perfusion map confirm the location of cancer in this case.

Some experts nearly demand the use of an endorectal coil for all of their patients, the main reason being the improved resolution and SNR, and therefore diagnostic confidence (Figure 3). This also has the advantage that there is no risk of confusion as to which protocol to choose, and if a patient initially planned for a detection scan subsequently gets scheduled for surgery, the same scan can be used for both indications. It can be frustrating if a scan optimized for detection in fact detects significant cancer, but cannot stage it because of technical limitations, making surgical treatment decisions less clear. One consideration for cases like this is to repeat* only* the T2WI with the endorectal coil, as the functional parameters are unlikely to be improved to a degree that they would affect the degree of suspicion for EPE. The drawback to this paradigm is that the patient must not only return for a second MRI, but that this additional scan might not be reimbursed. A consideration would be to bill for the endorectal coil only in this case.

### 5.2. Sensitivity and Specificity Differences

Looking at the literature for the use of mpMRI, it can be confusing given the myriad of listed sensitivity, specificity, and negative predictive values (NPV). Part of the reason is that the performance for cancer* detection* uses different criteria than for cancer* staging*. Another important factor is that the pretest probability will differ depending on how the group for evaluation is chosen, which is described in greater detail in Population Considerations Section.

A meta-analysis of 7 studies from 2007 through 2011 of a total of 526 men found a range of NPV ranging from 0.65 to 0.94 with specificity of 0.88 (95% CI, 0.82–0.92) and sensitivity of 0.74 (95% CI, 0.66–0.81) for prostate cancer detection [19]. A study from 1999 looking at T2WI and MRSI found specificity and sensitivity of 63–91% and 46–95%, respectively, in 53 men for both techniques combined, and since then mpMRI investigations with T2WI and some combination of DWI, DCE, and MRSI, looking at receiver-operating characteristic (ROC) curves, have found an area under the ROC curve (*A*_*z*_) ranging from 0.71 to 0.95 for prostate cancer detection [22, 23, 26, 27, 40–43]. At least one investigation has shown significant added value for each of the components of mpMRI, although some have shown less improvement for DCE, especially in the TZ [20, 26].

The performance for staging rather than detection of prostate cancer has focused more on sensitivity and specificity for EPE in the context of surgical planning. A meta-analysis in 2013 looking at 7 studies with a total of 603 subjects found a median sensitivity of 0.49 and specificity 0.82 for EPE with marked heterogeneity [44]. Although it is straightforward to determine the presence or absence of EPE, it is difficult to compare performance. Detection of EPE was at most a secondary endpoint of a number of studies, and many investigations analyzed low- and high-risk groups separately. In general, the sensitivity and specificity for EPE have ranged from 33–93% to 82–98%, respectively, with most articles reporting specificity in excess of 90% [2, 30, 32, 37, 45–47]. Those articles looking specifically at performance with or without an endorectal coil largely find improvement with the endorectal coil although not all authors found this to be significant [13, 14, 28, 48, 49].

### 5.3. Population Considerations

The difference in the relative performance of mpMRI for detection and staging, as described in the immediately preceding section, can be confusing initially but is likely in part explained by the difference in populations. A number of men referred for mpMRI optimized for detection will be “low risk,” with relatively low PSA and either no prior biopsy or prior biopsies showing no or only small volume, low-grade cancer. Compare this to the population of men referred for staging, who nearly all have significant cancer by biopsy. It is therefore expected that more diseases will be detected in the men referred for staging, in terms of both volume and aggressiveness. This informs not only the protocol, a more limited protocol is justified for detection as the pretest probability is much lower, but also the expectations of finding cancer at image review. It is quite rare that a scan done for staging will show no high-suspicion lesions, and were this the case, the scan should be carefully evaluated for technical limitations. However, depending on the series, half or more of scans done for detection will show no high-suspicion lesions [50].

**Figure 4:**
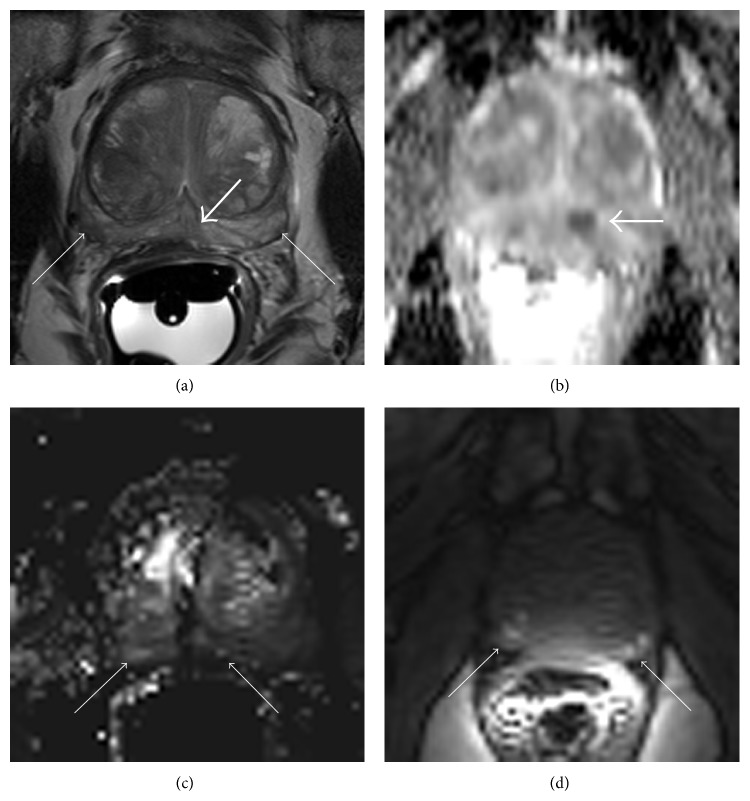
Hemorrhage can limit diagnostic confidence. The location of tumor (thick arrow) on T2-weighted imaging (a) is indistinct, with additional low signal areas in the peripheral gland bilaterally that appear mass-like (thin arrows). The location of tumor is confirmed on the apparent diffusion coefficient (ADC) map (b) but dynamic contrast-enhanced (DCE) perfusion (c) is heterogeneous (thin arrows). The precontrast T1-weighted image (d) reveals short T1 corresponding to the areas of low signal on T2-weighted imaging (thin arrows) confirming that this signifies artifact from hemorrhage.

Another important consideration is the presence of postbiopsy hemorrhage. Men referred for detection will normally have either prior negative biopsies or small-volume, low-grade disease and will not have had a biopsy for many months. The likelihood of hemorrhage is therefore low. However, when biopsy detects significant disease, staging is often the next course of action. The MRI scan may be ordered within a week or two of the biopsy when there may still be significant hemorrhagic artifact that could compromise diagnostic confidence (Figure 4). Although there is some indication that the degree to which hemorrhage compromises staging is negligible, it should still be more highly considered in this set of patients [51, 52].

## 6. Future Directions

The ESUR guidelines provide a framework for most clinical MRI centers to perform mpMRI at a level which can add value in terms of prostate cancer detection and staging. However, as described above, the performance even in expert hands can be quite variable. Improvements in T2WI focus primarily on spatial resolution or acceleration of acquisition [53]. However, there are countless ongoing investigations in all aspects of that which is currently considered mpMRI, including diffusion-tensor applications to DWI, more robust models for DCE, and faster or multidimensional MRSI [18, 54–56]. However, new techniques promise to add yet additional parameters to mpMRI, including elastography and hyperpolarized [1-^13^C]pyruvate [57, 58]. Combined with automated registration and presentation, and potentially automated detection, of prostate lesions, mpMRI is poised to go through a second revolution in patient management, leapfrogging our current understanding of prostate cancer biology [59]. Improvements in detection and characterization of prostate cancer hold the promise of nearly completely avoiding prostate biopsy, thereby avoiding the associated inflammation and scarring that can complicate surgery. It will also open the door to focal, less invasive therapies discussed in another paper in this special issue. The increase in patient throughput may bring the overall cost of mpMRI down, expanding its availability and potentially opening the door to a component of prostate cancer screening, or at least refining the suspicion for prostate cancer in men with elevated serum PSA.

## 7. Conclusions

The current implementation of mpMRI for the detection and staging of prostate cancer is now well established with a strong body of evidence proving its value and clear recommendations for its implementation. It has the potential of markedly improving the choice of treatment—if needed at all—in men with prostate cancer, allowing men with significant disease to get the right kind of treatment sooner while sparing unnecessarily aggressive treatment of potentially indolent disease. The adoption of mpMRI in everyday practice is, however, lagging, as expertise is not yet widespread. The groundswell of interest in mpMRI promises to change this in the near future.

## Figures and Tables

**Table 1:** Summary of technical recommendations from the 2012 ESUR guidelines.

Pulse sequence	Slice thickness		In-plane resolution		Parameters
	1.5 T	3.0 T	1.5 T	3.0 T	
T2WI	4 mm	3 mm	0.5 × 0.5 mm to 0.7 × 0.7 mm		2- or 3-plane
DWI	5 mm	4 mm	≤2.0 × 2.0 mm	≤1.5 × 1.5 mm	≥3 *b*-values, max *b*-value 1000 s/mm^2^
DCE	4 mm		1.0 × 1.0 mm	0.7 × 0.7 mm	≤15 s resolution over 5 min, 3 mL/s injection
MRSI	Optional, requires endorectal coil at 1.5 T, 0.5 cm^3^ voxel size, 8 × 8 × 8 phase encoding steps, field-of-view ≥1.5 voxels larger than volume of interest				
